# Facile and Eco-Friendly Synthesis of Finger-Like Co_3_O_4_ Nanorods for Electrochemical Energy Storage

**DOI:** 10.3390/nano5042335

**Published:** 2015-12-17

**Authors:** Shijiao Sun, Xiangyu Zhao, Meng Yang, Liqun Ma, Xiaodong Shen

**Affiliations:** College of Materials Science and Engineering, Nanjing Tech University, Nanjing 210009, China; E-Mails: sunshijiao@njtech.edu.cn (S.S.); yangmengyy@njtech.edu.cn (M.Y.); maliqun@njtech.edu.cn (L.M.); xdshen@njtech.edu.cn (X.S.)

**Keywords:** Co_3_O_4_ nanorods, electrochemical properties, lithium ion battery, supercapacitor

## Abstract

Co_3_O_4_ nanorods were prepared by a facile hydrothermal method. Eco-friendly deionized water rather than organic solvent was used as the hydrothermal media. The as-prepared Co_3_O_4_ nanorods are composed of many nanoparticles of 30–50 nm in diameter, forming a finger-like morphology. The Co_3_O_4_ electrode shows a specific capacitance of 265 F g^−1^ at 2 mV s^−1^ in a supercapacitor and delivers an initial specific discharge capacity as high as 1171 mAh g^−1^ at a current density of 50 mA g^−1^ in a lithium ion battery. Excellent cycling stability and electrochemical reversibility of the Co_3_O_4_ electrode were also obtained.

## 1. Introduction

One-dimensional nanomaterials have been attracting much attention for electrochemical energy storage, especially in a supercapacitor and a lithium ion battery [1,2,3]. Their high surface to volume ratio permits a high contact area with the electrolyte and hence more electroactive sites [4]. Moreover, the low dimension can enhance electron transport and ion diffusion. The time constant for diffusion is given by τ = *L*^2^/2*D*, where *L* is the diffusion length and *D* is the diffusion coefficient. *L* of the one-dimensional nanomaterials is decreased as compared with that of the bulk materials and accordingly a lower time constant is obtained [5,6]. In addition, nanomaterials can better accommodate the volume change during lithiation and delithiation [5].

Transition metal oxides which possess variable valence states have received considerable interest as electrodes in electrochemical energy storage systems, such as supercapacitors and the lithium ion battery. Cobalt oxides including binary oxides (CoO, Co_2_O_3_, and Co_3_O_4_) and ternary oxides (MCo_2_O_4_, M = transition metal) have been extensively studied. Compared with CoO or Co_2_O_3_, Co_3_O_4_ can be more easily prepared because many cobalt salts give Co_3_O_4_ upon heating in air at 300–400 °C [7]. For example, Co_3_O_4_ nanoparticles could be prepared by a molten salt method [8,9] or a urea combustion method [10]. For the application of Co_3_O_4_ in the lithium ion battery, the main problems include the large voltage hysteresis and amorphization upon discharge. Furthermore, cobalt is expensive and toxic. Partial substitution of Co with other abundant and/or eco-friendly elements to form ternary oxides MCo_2_O_4_ (M = Cu [11], Zn [12] or Mg [13]) has been performed in order to reduce the cost and toxicity of Co_3_O_4_. However, the reversible capacity of the ternary oxides usually degrades upon long-term cycling [7].

Up to now, one-dimensional Co_3_O_4_ nanomaterials with different architectures, such as nanotubes [4,14,15,16,17,18,19], nanorods [14,19,20,21,22,23,24], nanowires [25,26,27,28,29,30], nanobelts [20,31], nanofibers [32] and nanoneedles [33], have been prepared for electrochemical energy storage via various strategies. Nanorod is one of the most studied nanostructures. Several methods have been employed to synthesize Co_3_O_4_ nanorods. For example, Co_3_O_4_ nanorods with an average diameter of 20–50 nm and a length up to several micrometers were prepared via a complicated microwave-assisted hydrothermal method [24], in which a specially designed autoclave for the microwave heating is needed. Porous Co_3_O_4_ nanorods were prepared by a co-precipitation method, where argon protection is necessary, which also complicates the synthesis [23]. A microemulsion route was employed to prepare finger-like Co_3_O_4_ nanorods but unfortunately toxic cyclohexane and n-pentanol organic solvents were used [22].

Different from the above methods which are either complicate or toxic for the preparation of Co_3_O_4_ nanorods, herein, a facile hydrothermal strategy using H_2_O as solvent for the large-scale preparation of finger-like Co_3_O_4_ nanorods has been performed. These Co_3_O_4_ nanorods possess a length of several hundreds of nanometers. The electrochemical properties of the as-prepared Co_3_O_4_ nanorods as electrode material in the lithium ion battery or supercapacitor were investigated.

## 2. Results and Discussion

**Figure 1:**
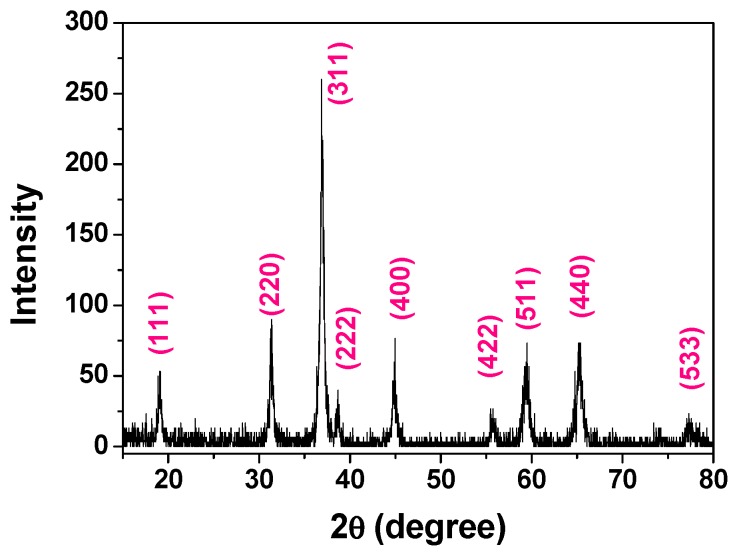
X-ray diffraction (XRD) pattern of the as-prepared Co_3_O_4_ nanorods.

Figure 1 shows the X-ray diffraction (XRD) pattern of the as-prepared Co_3_O_4_ material. All the diffraction peaks can be indexed to the pure cubic Co_3_O_4_ phase (JCPDS 65-3103). No impurity phase was detected. The average crystallite size of the as-prepared Co_3_O_4_ material calculated according to Scherrer’s formula is around 23 nm, which is smaller than that of the nanorods (48.2 nm) prepared via a gas bubble-assisted assembly method [19]. This small crystallite size can shorten the Li^+^ diffusion distance in the lithium ion battery.

**Figure 2:**
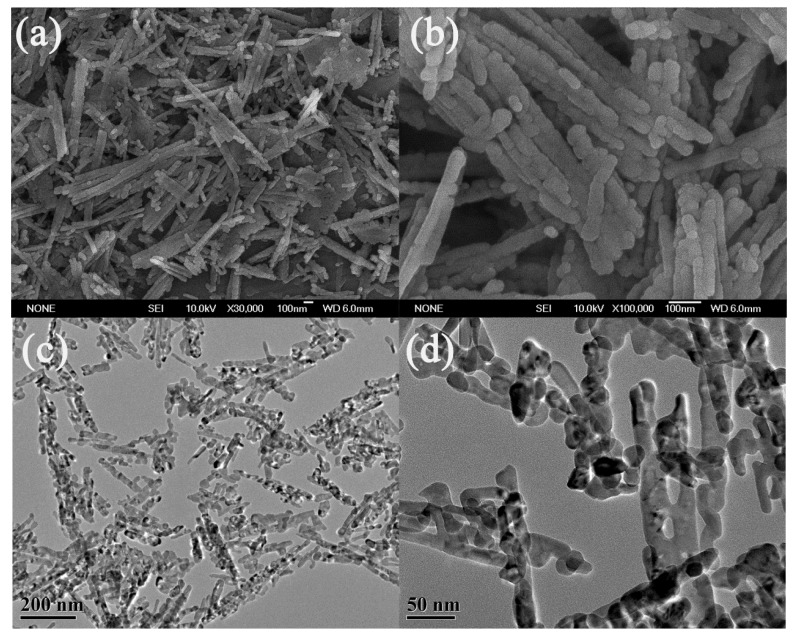
(**a**,**b**) Scanning electron microscopy (SEM) and (**c**,**d**) transmission electron microscopy (TEM) images of the as-prepared Co_3_O_4_ nanorods.

Figure 2 shows the scanning electron microscopy (SEM) and transmission electron microscopy (TEM) images of the as-prepared Co_3_O_4_ material. The low-magnification SEM image (Figure 2a) shows that the as-prepared Co_3_O_4_ material is composed of many nanorods of several hundreds of nanometers in length. Upon further magnification, we found these nanorods were formed by stacking many nanoparticles of 30–50 nm in diameter (Figure 2b), showing a finger-like morphology. TEM images (Figure 2c,d) confirm that each nanorod is composed of many aggregated nanoparticles. Mesopores are generated between adjacent nanoparticles, as shown in Figure 2d.

**Figure 3:**
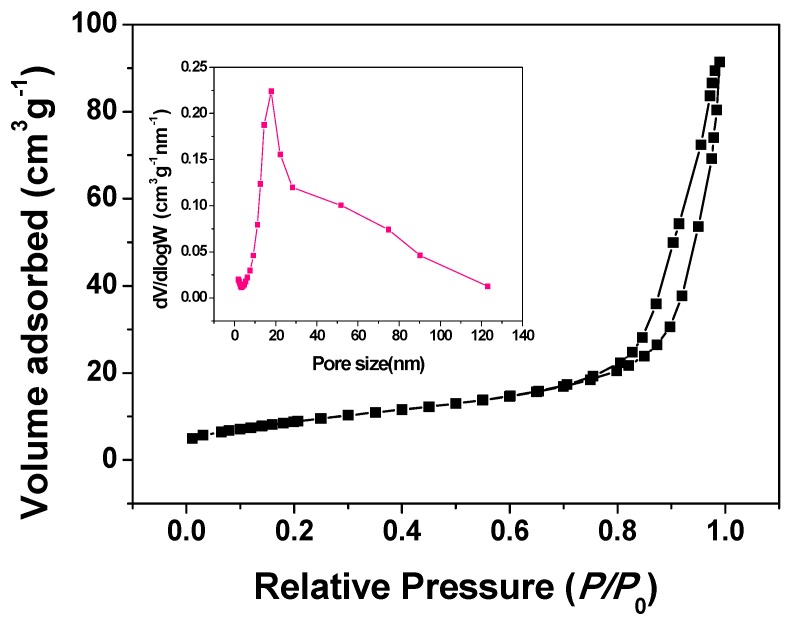
N_2_ adsorption-desorption isotherm and Barrett-Joyner-Halenda (BJH) pore size distribution (inset) curve of the as-prepared Co_3_O_4_ nanorods.

The N_2_ adsorption-desorption technique was used to characterize the porous structure of the as-prepared Co_3_O_4_ material. Figure 3 shows the isotherm and the corresponding Barrett-Joyner-Halenda (BJH) pore size distribution curve. The isotherm can be classified as mixed type II and type IV according to the unsaturated character at a partial pressure close to unity and the presence of a hysteresis loop, which are characteristics of the meso-macroporous materials [34]. The BJH pore size distribution curve exhibits a maximum value at around 18 nm. There, pores could be related to the interstices between the nanoparticles as shown in the TEM image in Figure 2d. The calculated average pore size, Brunauer-Emmett-Teller (BET) surface area, and pore volume are 15 nm, 33 m^2^ g^−1^ and 0.11 cm^3^ g^−1^, respectively. The surface area of the as-prepared Co_3_O_4_ material is smaller than the reported values of 95.2 m^2^ g^−1^ and 115.27 m^2^ g^−1^ for the Co_3_O_4_ nanorods prepared by the co-precipitation [23] and microemulsion [22] methods, respectively. While it is much larger than 0.31–19.4 m^2^ g^−1^ for the Co_3_O_4_ nanoparticles prepared by the molten salt method [8,9]. The porous structure of our finger-like Co_3_O_4_ nanorods may facilitate electrolyte penetration and provide spaces for the strain release in both the supercapacitor and lithium ion battery systems.

**Figure 4:**
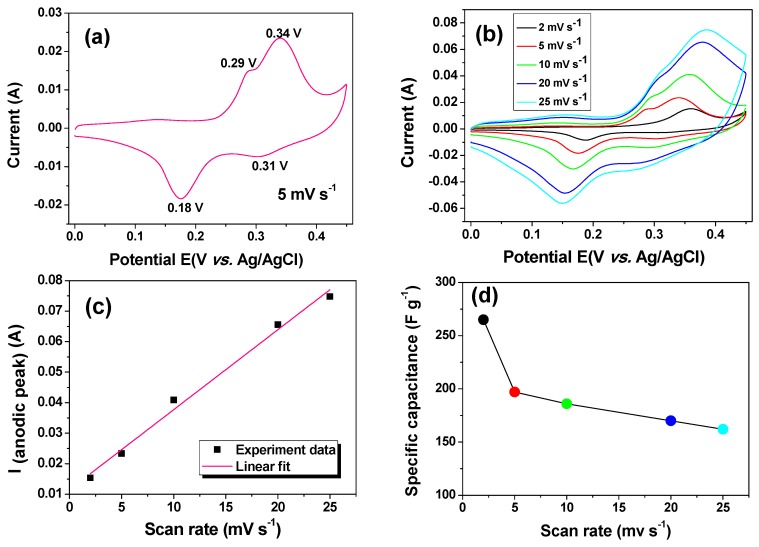
Capacitive performance of the as-prepared Co_3_O_4_ nanorods in a potential range of 0–0.45 V. (**a**) Cyclic voltammetric (CV) curve at a scan rate of 5 mV s^−1^; (**b**) CV curves at different scan rates; (**c**) peak current as a function of scan rate from (**b**); (**d**) rate dependent specific capacitance.

The capacitive performance of the as-prepared Co_3_O_4_ electrode was evaluated by cyclic voltammetry (CV). Figure 4a shows the CV curve at a scan rate of 5 mV s^−1^ in a potential range of 0–0.45 V. Two pairs of redox peaks (0.29/0.18 V and 0.34/0.31 V) were observed. The redox couple located at 0.29/0.18 V corresponds to the conversion reaction between CoOOH and Co_3_O_4_. The other couple located at 0.34/0.31 V is related to the conversion reaction between CoOOH and CoO_2_ [30]. The CV curves at different scan rates are shown in Figure 4b. With the increase of the scan rate, the current response increases. A quasi-linear relationship is observed between the redox peak current and the scan rate (Figure 4c), indicating a diffusion-controlled process and dominant surface redox reactions [35,36]. These characteristics are typical for a pseudocapacitor. The calculated specific capacitances at scan rates of 2, 5, 10, 20, and 25 mV s^−1^ are 265, 197, 186, 170, and 162 F g^−1^, respectively. The specific capacitance of the as-prepared finger-like Co_3_O_4_ nanorods is higher than the reported value of the needle-like Co_3_O_4_ nanorods (111 F g^−1^) [37], although our sample has a lower surface area. The unique finger-like morphology of our Co_3_O_4_ material is responsible for its better performance.

**Figure 5:**
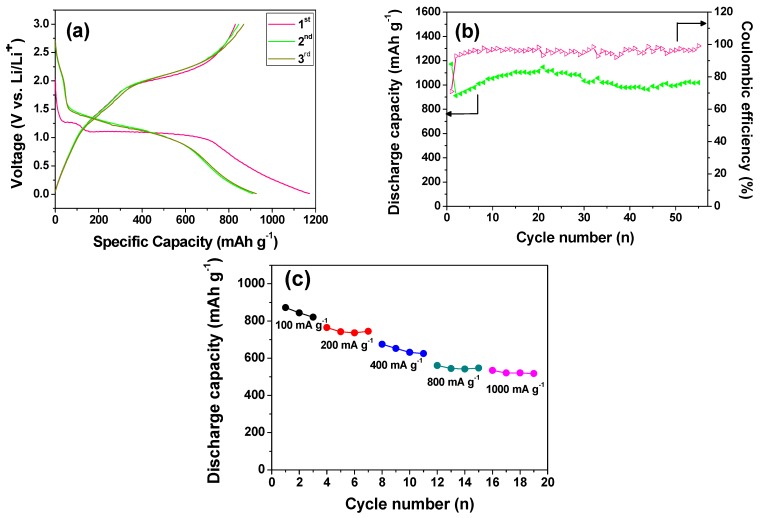
The lithium storage performance of the as-prepared Co_3_O_4_ nanorods at a current density of 50 mA g^−1^. (**a**) The first three charge-discharge curves; (**b**) cycling performance; (**c**) rate performance.

The as-prepared Co_3_O_4_ nanorods were further tested as the anode in a lithium ion battery. Figure 5a shows the first three charge (delithiation) and discharge (lithiation) curves at a current density of 50 mA g^−1^ between 0.01 and 3.0 V. The first discharge profile displays a small plateau at around 1.25 V and a distinct plateau at around 1.0 V. The small plateau at 1.25 V corresponds to the intercalation reaction by the formation of Li*_x_*Co_3_O_4_ when nanosized Co_3_O_4_ anode material was used [7,9]. The subsequent discharge results in the destruction of the Li*_x_*Co_3_O_4_ structure and the formation of Co metal nanoparticles and Li_2_O. This is responsible for the distinct plateau at 1.0 V. The as-prepared Co_3_O_4_ nanorods deliver an initial discharge capacity as high as 1171 mAh g^−1^. In the first charge process, a voltage plateau at around 2.1 V is observed, which could be attributed to the reversible oxidation of Co to cobalt oxide. Besides, the discharge profiles in the subsequent cycles are different in shape from that in the first cycle. This is similar to previous reports [4,38,39,40,41,42]. The discharge capacity and coulombic efficiency as a function of the cycle number are shown in Figure 5b. The as-prepared Co_3_O_4_ nanorod electrode demonstrates excellent cycling capability. The increase of the discharge capacity in the initial cycles may be due to the gradual establishment of the Co/Li_2_O interface for extra Li storage, *i.e.*, an activation process [38]. After 50 cycles, the discharge capacity still maintains 1006 mAh g^−1^, which is comparable with or even higher than the reported values for other forms of Co_3_O_4_ nanostructures [19,43,44,45,46,47,48]. Moreover, the coulombic efficiency maintains around 95% since the second cycle, indicates the excellent electrochemical reversibility of the as-prepared Co_3_O_4_ nanorod electrode. This superior electrochemical performance can be ascribed to its unique nanostructure. As the as-prepared Co_3_O_4_ nanorods are composed of many nanoparticles, the number of boundaries between adjacent nanoparticles is large. Correspondingly, compared with a perfect nanorod, the as-prepared Co_3_O_4_ nanorod is more thermodynamically unstable and many more electroactive sites are generated. Note that the specific discharge capacities of the finger-like Co_3_O_4_ nanorods are much higher than the theoretical capacity of Co_3_O_4_ (890 mAh g^−1^). The extra capacity may be ascribed to the lithium storage at the Co/Li_2_O interface [48]. Furthermore, the rate capability of the as-prepared Co_3_O_4_ nanorods was studied, as shown in Figure 5c. When the current density increases from 100 to 1000 mA g^−1^, the specific discharge capacity decreases from 872 to 518 mAh g^−1^.

**Figure 6:**
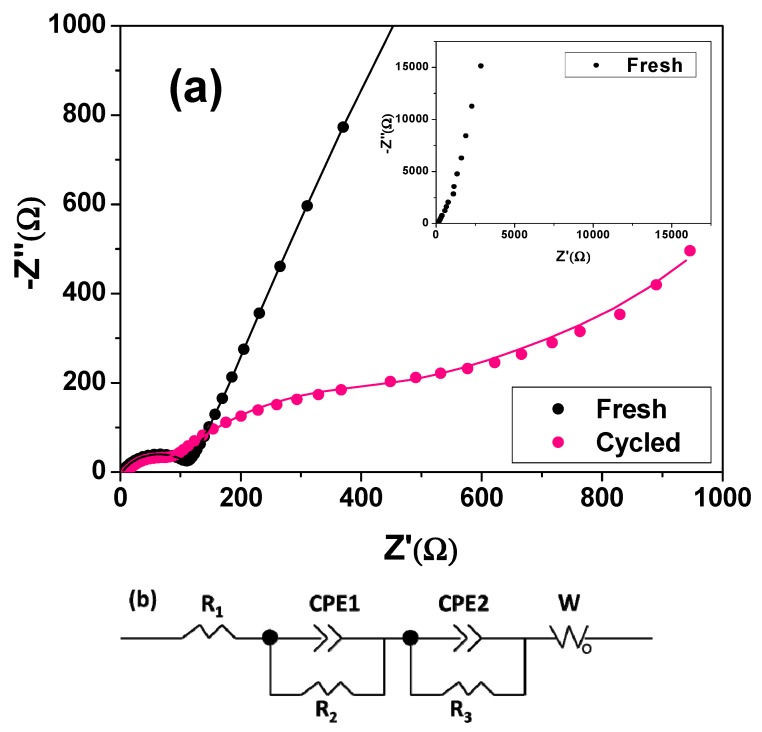
(**a**) Enlarged Nyquist plot of the fresh Co_3_O_4_ nanorod electrode at high frequency and Nyquist plot of the cycled electrode in the whole frequency region; the dot and the line are related to the experimental and fitting results, respectively; the inset is the Nyquist plot of the fresh electrode in the whole frequency region. (**b**) Equivalent circuit for the fresh and cycled electrodes; *R*_1_ represents the solution resistance (*R*_s_) for both electrodes; *R*_2_ is the contact resistance (*R*_c_) for the fresh electrode or the resistance caused by the SEI layer (*R*_SEI_) for the cycled electrode; *R*_3_ corresponds to the Li^+^ charge-transfer resistance (*R*_ct_); CPE1 and CPE2 are constant phase elements; W represents the Warburg resistance.

The electrochemical impedance spectroscopy (EIS) technique was used to investigate the charge transfer in the electrode material. Figure 6a shows the Nyquist plots of the fresh and cycled Co_3_O_4_ electrodes measured at open circuit voltage over a frequency range from 10^5^ Hz to 0.01 Hz with an amplitude of 5 mV. For both electrodes, a depressed semicircle in the high-frequency region, a broad arc in the medium-frequency region and an inclined line in the low-frequency region were observed. The broad arc for the fresh electrode is visible only when the plot is presented in the whole frequency range (inset in Figure 6a) and more obvious from the corresponding bode plot (Figure S1). The equivalent circuit model shown in Figure 6b was used to fit the Nyquist plots of both electrodes. The semicircles at the high frequency region for the fresh and cycled electrodes represent the contact resistance (*R*_c_) or the resistance caused by the SEI layer (*R*_SEI_). The arc at the medium frequency region and the inclined line at the low frequency correspond to the Li^+^ charge-transfer resistance (*R*_ct_) on the electrode/electrolyte interface and the Warburg resistance (*W*), respectively. The diameter of the arc represents the value of *R*_ct_. The Warburg resistance reflects the solid-state diffusion of Li ions in the bulk electrode [43]. Constant phase elements (CPE1 and CPE2) rather than the pure capacitance (*C*) reflect the non-homogeneous nature of the electrode, which leads to the formation of a depressed semicircular shape [49]. The fitting results are shown in Table 1. Consistent with previous reports [46,50], the charge-transfer resistance of the cycled electrode (215 Ω) is much smaller than that of the fresh electrode (695 Ω). The conversion reaction at the Co_3_O_4_ anode leads to the generation of many new grain boundaries and interfaces, which would facilitate the charge transfer reaction upon cycling. Moreover, the charge-transfer resistance of the cycled electrode herein is comparable with those of Co_3_O_4_ nanoparticles with opened-book morphology [44] and mesoporous Co_3_O_4_ nanoflakes [51].

**Table 1:** Impedance parameters based on the equivalent circuit above for both the fresh and cycled Co_3_O_4_ electrodes.

Resistance (Ω)	Fresh Electrode	Cycled Electrode
*R*_s_	3.74	9.02
*R*_c_ or *R*_SEI_	111	42
*R*_ct_	695	215

## 3. Experimental Section

### 3.1. Sample Preparation and Characterization

All chemicals were purchased from Shanghai Chemical Regent Co. (Shanghai, China) with analytical grade and used without further purification. Co_3_O_4_ nanorods were prepared via a facile and eco-friendly hydrothermal route. Typically, 1.99 g of cobalt acetate tetrahydrate (C_4_H_6_CoO_4_·4H_2_O), 1.92 g of urea and 1.00 g of surfactant cetyltrimethylammonium bromide (CTAB) were dissolved in 100 mL deionized water to give a transparent solution. The obtained solution was loaded into a Teflon-lined stainless steel autoclave, which was sealed and heated at 100 °C for 24 h in an oven. The system was then naturally cooled to room temperature, and a pink precipitation was collected. The product was washed with distilled water and anhydrous alcohol, and dried at 60 °C. Finally, the product was calcined at 400 °C for 2 h in a muffle furnace in air.

The crystal structure of the as-prepared Co_3_O_4_ material was examined by powder X-ray diffraction (XRD) (Rigaku D/MAX 2200, using Ni-filtered Cu Ka radiation, Rigaku Corporation, Tokyo, Japan). The morphology of the as-prepared Co_3_O_4_ material was investigated by field emission scanning electron microscopy (FESEM) (JEOL JSM-6700F, operating at 20 kV, JEOL Ltd., Tokyo, Japan) and transmission electron microscopy (TEM) (JEOL JEM-2010, operating at 200 kV, JEOL Ltd., Tokyo, Japan). Nitrogen adsorption-desorption isotherm was performed at 77 K using a Micromeritics ASAP 2020M analyzer (Micromeritics Corporate, Norcross, GA, USA). Prior to determination of the isotherm, the sample was degassed at 150 °C for 5 h under vacuum. The Brunauer-Emmett-Teller (BET) specific surface area was calculated using the adsorption data in the relative pressure (*P*/*P*_0_) range of 0.05 to 0.3, and the total pore-volume was determined from the amount adsorbed at *P*/*P*_0_ = 0.98. The pore-size distribution curve was calculated based on the desorption branch of the isotherm using the Barrett-Joyner-Halenda (BJH) method.

### 3.2. Electrochemical Characterization

For capacitive measurement, the working electrode containing 7 mg of the active material was prepared according to the following steps. 70 wt % as-prepared Co_3_O_4_, 25 wt % acetylene black as a conducting agent and 5 wt % polytetrafluoroethylene (PTFE) as a binder were homogeneously mixed and pressed onto a nickel foam (1.5 cm × 1.5 cm) current collector under a pressure of 12 MPa. The capacitive performance was evaluated in 6 M KOH electrolyte using a three electrode experimental setup. The as-prepared electrode, platinum sheet and Ag/AgCl electrode were used as working, counter, and reference electrodes, respectively. Cyclic voltammetric (CV) measurement was conducted using a CHI440 Electrochemical Workstation (CH Instruments Inc., Austin, TX, USA).

For lithium storage measurement, a working electrode containing 1–2 mg of the active material was constructed according to the following steps. 75 wt % as-prepared Co_3_O_4_, 15 wt % acetylene black as a conductive agent and 10 wt % polyvinylidene fluoride (PVDF) as a binder were homogeneously mixed in *N*-methyl-2-pyrrolidone (NMP) solvent. The obtained paste was cast onto Cu foil. After evaporation of the solvent at 80 °C for 2h, the electrode was punched into disks of 14 mm in diameter. The electrode was further dried at 100 °C under vacuum for 12 h. The lithium storage performance was evaluated using a two-electrode CR2032-type coin cell. The electrolyte was 1 M LiPF_6_ in ethylene carbonate (EC) and dimethyl carbonate (DMC) (1:1 *w*/*w*). Metallic lithium was used as both counter and reference electrodes. Cell assembly was operated in an argon-filled glove box. Galvanostatic and electrochemical impedance measurements were conducted on a LAND CT2001A battery test system (Landt Instrument, Wuhan, China) and an Autolab electrochemical workstation (Metrohm Autolab, Utrecht, The Netherlands), respectively.

All the electrochemical measurements were conducted at ambient temperature.

## 4. Conclusions

Finger-like Co_3_O_4_ nanorods were prepared by a facile and eco-friendly hydrothermal method. The as-prepared Co_3_O_4_ nanorods consist of many nanoparticles of 30–50 nm in diameter, forming a unique finger-like architecture. Moreover, these unique Co_3_O_4_ nanorods exhibited excellent electrochemical performance for the supercapacitor and lithium ion battery. The initial discharge capacity at the current density of 50 mA g^−1^ is as high as 1171 mAh g^−1^. After 50 cycles, the discharge capacity still maintains 1006 mAh g^−1^, which is comparable with or even higher than the reported values on other Co_3_O_4_ nanomaterials. The as-prepared finger-like Co_3_O_4_ nanorods show great potential for application in the lithium ion battery.

## Supplementary Materials

Supplementary materials can be accessed at: http://www.mdpi.com/2079-4991/5/4/2335/s1.
