# Supplementary material for: Facile and Eco-Friendly Synthesis of Finger-Like Co3O4 Nanorods for Electrochemical Energy Storage
Source: Nanomaterials (Basel). 2015 Dec 17;5(4):2335–47. doi: 10.3390/nano5042335 (PMC5304806; doi:10.3390/nano5042335)
Supplement: Supplementary file 1 [file nanomaterials-05-02335-s001.pdf]

Supplementary Information

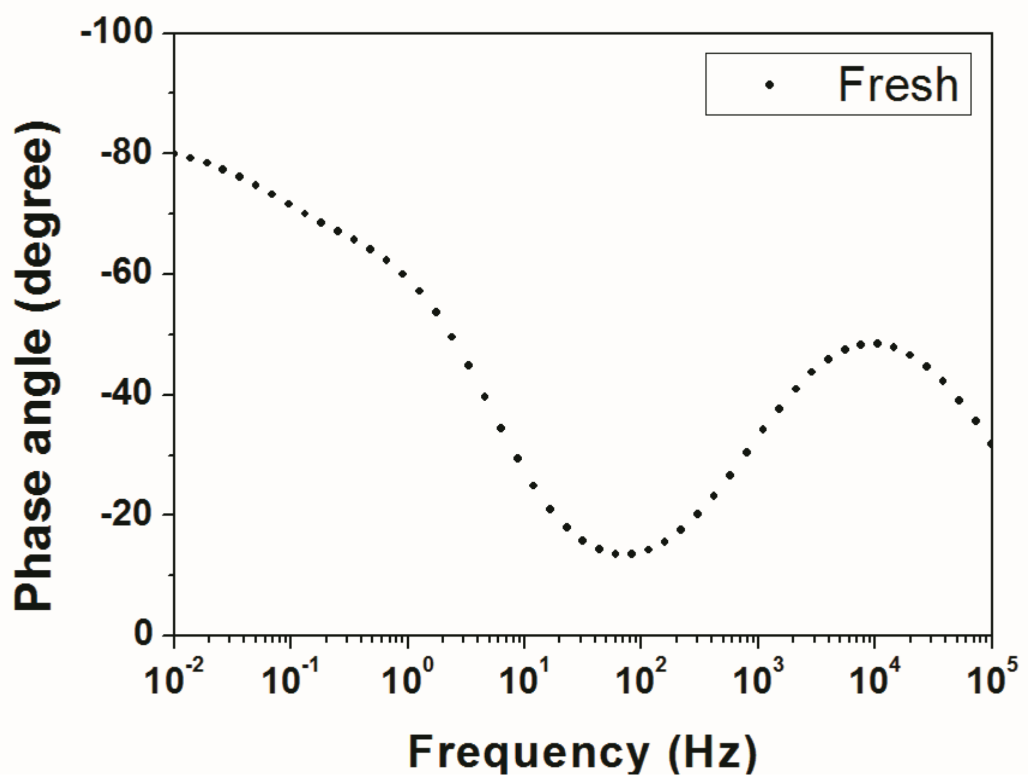

Figure S1. Bode plot of the fresh Co_3O_4 nanorod electrode.

© 2015 by the authors; licensee MDPI, Basel, Switzerland. This article is an open access article distributed under the terms and conditions of the Creative Commons Attribution license (http://creativecommons.org/licenses/by/4.0/).
